# Evaluation of antimicrobial susceptibility of *Escherichia coli* strains isolated in Rabat University Hospital (Morocco)

**DOI:** 10.1186/s13104-015-1380-9

**Published:** 2015-08-30

**Authors:** Nabil Alem, Mohammed Frikh, Abdellatif Srifi, Adil Maleb, Mariama Chadli, Yassine Sekhsokh, Lhoucin Louzi, Azzedine Ibrahimi, Abdelhay Lemnouer, Mostafa Elouennass

**Affiliations:** Hôpital Militaire d’Instruction Mohammed V, Rabat, Morocco; Faculté de Médicine et de Pharmacie de Rabat Université Mohammed V Souissi, Rabat, Morocco; Faculté de Médecine de Pharmacie d’Oujda Université Mohammed I, Oujda, Morocco; villa 96 la corniche hay el fath, Rabat, Morocco

**Keywords:** *Escherichia coli*, Antimicrobial susceptibility, Morocco

## Abstract

**Background:**

*Escherichia coli* (*E. coli*) is the most commonly isolated bacteria in human pathology. In Morocco the data concerning the nature and the rates of antibiotic resistance of *E. coli* in both hospitals and city environment remains relatively poor and needs further investigations.

**Methods:**

During a 16 months period, *E. coli* isolates were collected from different culture specimens received in the Bacteriology Department of the Military teaching Hospital Mohammed-V-Rabat for routine diagnostic purposes. *E. coli* isolates were identified and their antimicrobial susceptibility pattern was determined.

**Results:**

A total of 1369 *E. coli* isolates comprising 33 % (1369/4110) of culture-positive samples were consecutively collected. Isolates of *E. coli* were, in 40.5 % (554/1369) of cases from hospitalized patients and in 59.5 % (815/1369) of cases from outpatients. Urine isolates represented 82 % (1123/1369) of the cases. High rates of resistance were found for amoxicillin (42.5 %), cefalotin (30.4 %), norfoloxacin (29.9 %) and sulfamethoxazole (37.7 %). The detection rate of ESBL was 6.1 % (85/1369). In hospitalized patients 11.9 % of the isolates of *E. coli* (66/554) had an ESBL phenotype while in outpatients cases only 2.3 % of isolates of *E. coli* (19/815) had this phenotype.

**Conclusions:**

Our findings suggest that more judicious use of antibiotics is needed especially in probabilistic treatment. The emergence of ESBL in the Moroccan cities is an indicator of the severity of this problem that is not limited to health care facilities.

## Background

*Escherichia coli* (*E. coli*) is a common commensal of the intestine of humans and animals but can also be found in water, soil and vegetation [1]. It is the most frequently isolated bacteria from clinical samples [1], indeed *E. coli* is the pathogen most involved in urinary tract infections [2–4] and one of the common agents responsible for ear infections, sepsis and wound infections [5, 6]. In the developing countries, *E. coli* represents the agent most commonly responsible for food and waterborne diarrhea and causes high mortality in children under 5 years old [7].

*Escherichia coli* dominates the overall spectrum of the bacterial infections in both hospitals and the community [8]. Therefore its susceptibility to antibiotics reflects both the hospital and community antibiotic selection pressure [9].

The emergence of resistance is a global phenomenon, although the rates of antibiotic resistance remain different between developed and developing countries [10, 11]. This emergence complicates the management of infections and impacts the use of widely prescribed antibiotics in clinical practice such as penicillins, sulfonamides and fluoroquinolones.

The aim of this study is to determine the resistance rate of *E. coli* isolates to different antibiotics in the Rabat Military Teaching Hospital Mohammed V and to compare these rates of resistance between hospitalized patients and outpatients and between urine isolates and other isolates.

## Methods

### Materials

The present study was conducted in the bacteriology department of the Rabat Military Teaching Hospital Mohammed V. Retrospectively from April 1, 2012 to July 31, 2013. We included all *E. coli* isolates received from hospitalized patients and outpatients. We highlight that under Moroccan law no ethical approval is required for a retrospective study based on laboratory data and no consent from patients is necessary to carry out further tests on samples collected for other purposes.

In order to eliminate duplicates, only one strain isolated from the same patient with the same antibiotic susceptibility was included.

### Bacterial identification and antimicrobial susceptibility

The identification of bacterial isolates was based on cultural, morphological and biochemical characteristics. Biochemical identification was set up using API20E (bio-Mérieux SA, Marcy-l’Étoile/France).

Antibiotic susceptibility was determined using the agar diffusion method (Mueller–Hinton medium) and its interpretation was made according to the recommendations of antibiogram committee of the French Society for Microbiology [12].

Antibiotics tested were: amoxicillin–clavulanic acid, cephalothin, cefoxitin, ceftriaxone, ertapenem, gentamicin, amikacin, norfoloxacin, cotrimoxazole and fosfomycin.

Detection of extended β-lactamases (ESBL) was performed by phenotypic method based on the detection of synergy between amoxicillin–clavulanic acid disc and three discs of third-generation cephalosporins: cefotaxim, ceftazidime and cefepime [12].

### Statistical analysis

The Chi square test was used to compare resistance rates. The difference between the frequencies was considered to be significant when *p* was <0.05.

## Results

During the period of our study, were received at the bacteriology department a total of 32,522 samples. The sex ratio male/female was 1.03 (16,515/16,007). The rate of urine samples was 25.09 % (8159/32,522) with a sex ratio male/female 0.73 (3459/4700). The overall incidence of isolation of *E. coli* was 33.3 % (1369/4110). The sex ratio male/female was 0.65 (538/830). Isolates of *E. coli* were in 40.5 % (554/1369) of cases from hospitalized patients and in 59.5 % (815/1369) of cases from outpatients. Urine isolates represented 82 % (1123/1369) of the cases.

Isolates with ESBL phenotype represented 6.1 % of all *E. coli* isolates (85/1369). In hospitalized patients 11.9 % of the isolates of *E. coli* (66/554) had an ESBL phenotype while in outpatients cases only 2.3 % of isolates of *E. coli* (19/815) had this phenotype. Furthermore 84.4 % (43/66) of the isolates with ESBL phenotype in hospitalized patients were also resistant to fluoroquinolones (FQ resistance + ESBL).

High rates of resistance were found for amoxicillin–clavulanic acid (42, 5 %), cefalotin (30, 4 %), norfoloxacin (29, 9 %) and cotrimoxazole (37, 7 %).

Table 1 shows the rates of resistance (R + I) within *E. coli* isolates depending on the nature of patients. Frequencies of resistance among isolates from hospitalized patients were higher than those from outpatients in the case of: amoxicillin–clavulanic acid (p < 0.001), cephalothin (p < 0.001), cefoxitin (p = 0.001), ceftriaxone (p < 0.001), amikacin (p = 0.001) and norfoloxacin (p = 0.003).

**Table 1:** Rates of resistance (R + I) of *Escherichia coli* isolates depending on the nature of patients

	Isolates *n*	Out patients (*R* + *I*)		Hospitalized patients (*R* + *I*)		*p*
		*n*	%	*n*	%	
Amoxicillin/clavulanic ac (AMC)	1263	286	38.0	246	48.1	<0.001
Cefalotin (KF)	1263	196	26.1	185	36.2	<0.001
Cefoxitin (FOX)	1256	22	2.9	34	6.7	0.001
Ceftriaxone (CRO)	1032	28	4.0	32	9.8	<0.001
Ertapenem (ERT)	1251	14	1.9	17	3.4	0.098
Gentamicin (GEN)	1137	66	9.8	63	13.6	0.044
Amikacin (AK)	1250	4	0.5	14	2.8	0.001
Norfoloxacine (NOR)	1020	182	26.1	113	35.1	0.003
Cotrimoxazole (SXT)	1244	261	35.2	195	38.8	0.203
Fosfomycin (FOS)	1082	13	2.0	9	2.1	0.974

Table 2 shows the rates of resistance (R + I) of *E. coli* isolates depending on the nature of sample. Frequencies of resistance among isolates from urine samples were lower than those from others samples in the case of: amoxicillin–clavulanic acid (p < 0.001), cephalothin (p < 0.001), cefoxitin (p = 0.004), ceftriaxone (p < 0.001), gentamicin (p < 0.001) and amikacin (p < 0.001).

**Table 2:** Rates of resistance (R + I) of *Escherichia coli* isolates depending on the nature of samples

	Urine (*R* + *I*)		Other samples (*R* + *I*)		*p*
	*n*	%	*n*	%	
Amoxicillin/clavulanic ac (AMC)	422	38.8	135	56.7	<0.001
Cefalotin (KF)	292	26.9	103	43.1	<0.001
Cefoxitin (FOX)	40	3.7	19	8.0	0.004
Ceftriaxone (CRO)	60	5.6	50	21.3	<0.001
Ertapenem (ERT)	26	2.4	4	1.7	0.516
Gentamicin (GEN)	90	9.2	38	17.7	<0.001
Amikacin (AK)	7	0.7	10	4.2	<0.001
Norfoloxacine (NOR)	313	29.1	74	31.2	0.52
Cotrimoxazole (SXT)	398	37.2	92	38.8	0.648
Fosfomycin (FOS)	18	1.9	4	2.1	0.779

The rate of simultaneous resistance to all of the three antibiotics which are most used orally (AMC + SXT + FQ) was 8.3 % in isolates from hospitalized patients compared to 9.9 % outpatients (*p* = 0.34).

## Discussion

In our study *E. coli* represented over a third of the total isolates of our department. Urinary tract remains the main site of colonization-infection totaling about 82 % of all isolates. These proportions are similar to those found in French and European epidemiological studies [13, 14].

We recorded important levels of resistance (R + I) for amoxicillin–clavulanic acid (38 % in outpatients and 48.1 % in hospitalized patients) these percentages of resistance are comparable to those of Onerba-France—with 36 % in city and 45 % in hospital [15]. Rates of resistance (R + I) for AMC in hospitalized patients were higher than those from outpatients (p < 0.001).

ESBL and resistance to fluoroquinolones are the two most worrying phenomena [16]. A review of Moroccan data shows varying levels of frequency of ESBL by region, structures and the size of the populations studied. These rates vary between 7 and 15 % [17, 18].

Our study showed a 12.4 % rate of ESBL *E. coli* in hospitalized patients, this rate remains similar to that recorded by a recent study in Rabat [17] and lower than the one recorded in Khartoum-Soudan [8]. ESBL is not limited to health care facilities; international studies show that in community setting rates of ESBL *E. coli* range from 1.3 to 4.8 % [19–21]. We recorded a 2.5 % rate of ESBL *E. coli* in our outpatients.

Fluoroquinolone resistance is associated with the misuse of these molecules in human and veterinary medicine [22]. This resistance varies from one geographic area to another with 10 % in France and United States vs. 40 % in China [15, 23, 24].

Our study found a rate of resistance of *E. coli* to fluoroquinolones 29.9 % which is similar to that recorded in Rabat [17] with a frequency of resistance among isolates from hospitalized patients higher than those from outpatients (p = 0.003).

A frequent association between genetic determinants of Qnr and those of ESBL was reported by several studies [25]. In our study we found 43 multiresistant strains (ESBL + FQ resistance), more genetic studies are needed to characterize the nature of fluoroquinolone resistance determinants carried by these strains.

The rate of resistance to aminoglycosides remains relatively low (gentamicin 11.1 %, amikacin 1.3 %) as reported in the literature [8]. Amikacin appears to be the most effective molecule of this class of antibiotics explained by the fact that it is strictly used in hospitals and is rarely used in the first line therapy.

In our study, fosfomycin remains largely active on isolates of *E. coli* with low resistance rates especially among urine isolates (1.9 %). These results suggest that we should favor the use of fosfomycin as a molecule for the empirical treatment of community urinary infections.

## Conclusion

Periodic monitoring of antibiotic resistance in different bacterial isolates has become essential given the constant evolution of the bacterial ecology and the emergence of antibiotic resistance. The high rate of multiresistance shown in this study should encourage us to be more judicious in the use of antibiotics especially in probabilistic treatment. Indeed the 10 % threshold of resistance is substantially exceeded for several antibiotics used in our hospital. The emergence of ESBL in the community is an indicator of the seriousness of this problem which appears not to be limited to health care facilities.

## Abbreviations

AMC: amoxicillin/clavulanic ac
AK: amikacin
CRO: ceftriaxone
*E. coli*: *Escherichia coli*
ERT: ertapenem
ESBL: extended spectrum beta lactamase
FQ: fluoroquinolon
FOS: fosfomycin
FOX: cefoxitin
GEN: gentamicin
KF: cefalotin
NOR: norfoloxacin
Onerba: Observatoire National de l’Epidémiologie de la Résistance
SXT: cotrimoxazole
